# Fast and Precise 3D Fluorophore Localization based on Gradient Fitting

**DOI:** 10.1038/srep14335

**Published:** 2015-09-22

**Authors:** Hongqiang Ma, Jianquan Xu, Jingyi Jin, Ying Gao, Li Lan, Yang Liu

**Affiliations:** 1 Biomedical and Optical Imaging Laboratory, Departments of Medicine and Bioengineering, University of Pittsburgh, Pittsburgh PA 15213, USA; 2 School of Medicine, Tsinghua University, China; 3 Department of Microbiology and Molecular Genetics, University of Pittsburgh, Pittsburgh PA 15213, USA

## Abstract

Astigmatism imaging approach has been widely used to encode the fluorophore’s 3D position in single-particle tracking and super-resolution localization microscopy. Here, we present a new high-speed localization algorithm based on gradient fitting to precisely decode the 3D subpixel position of the fluorophore. This algebraic algorithm determines the center of the fluorescent emitter by finding the position with the best-fit gradient direction distribution to the measured point spread function (PSF), and can retrieve the 3D subpixel position of the fluorophore in a single iteration. Through numerical simulation and experiments with mammalian cells, we demonstrate that our algorithm yields comparable localization precision to the traditional iterative Gaussian function fitting (GF) based method, while exhibits over two orders-of-magnitude faster execution speed. Our algorithm is a promising high-speed analyzing method for 3D particle tracking and super-resolution localization microscopy.

Localization microscopy, known as different names including (fluorescence) photo-activated localization microscopy [(f) PALM]^1^,^2^ and (direct) stochastic optical reconstruction microscopy [(d) STORM]^3^,^4^, has become a powerful imaging tool to reveal the ultra-structures and understand the complicated mechanisms behind cellular function. The principle of localization microscopy is straightforward: a small subset of densely labeled fluorophores is sequentially switched “on” to obtain the sparsely distributed individual fluorescent emitters in a single frame, and the position of each emitter is determined by localization algorithm at a nanometer precision; after accumulating the localized positions from thousands of imaging frames, the spatial resolution of the final reconstructed image can be improved by ~10 times.

By further combining the point spread function (PSF) engineering methods^5^,^6^,^7^, the capabilities of localization microscopy have been extended to resolve biological structures in all three dimensions. Various PSF engineering methods share a similar underlying principle that the axial position is encoded as the shape of the PSF in the lateral plane, which can be later decoded through image analysis. Among them, astigmatism approach has gained popularity because of its simple experimental configuration. By introducing astigmatism to the optical system (using a cylindrical lens^5^,^8^,^9^ or deformable mirror^10^,^11^), the axial position of the fluorophore is encoded as the ellipticity of the PSF. Generally, by employing a 2D elliptical Gaussian function to fit the elliptical PSF, a resolution of ~20 nm in the lateral dimension and ~50 nm in the axial dimension have been achieved^5^.

The spatial resolution of localization microscopy is directly affected by the precision of the localization algorithm. For the best spatial resolution, iterative Gaussian function fitting (GF) based algorithms are usually employed^12^,^13^. But the slow execution speed of such algorithm that often takes several hours to reconstruct a standard super-resolution image is an intrinsic disadvantage of the GF based methods. Hence, they do not apply to the cases when fast image reconstruction and online data analysis are needed, such as real-time optimization of imaging parameters. For this purpose, several single-iteration algorithms have been developed in the past few years to accelerate the execution speed while providing comparable precision to the GF based algorithm^14^,^15^,^16^,^17^,^18^. Unfortunately, these algorithms are mainly designed for 2D fitting of a circular PSF, and their precision for retrieving the 3D position is significantly compromised when the spatial distribution of fluorescent emission is not isotropic, such as astigmatism-based imaging with elliptical PSF. Hence, it is important to develop a highly efficient 3D localization algorithm for astigmatism-based single particle tracking or super-resolution localization microscopy with both satisfactory localization precision and execution speed.

In this paper, we present an algebraic algorithm based on gradient fitting for fast 3D fluorophore localization in astigmatism-based microscopy. We utilize the relationship of the gradient direction distribution and the position of the fluorescent emitter to determine the x–y position and ellipticity of the PSF by finding the best-fit gradient direction distribution (Fig. 1a). Then, this algorithm estimates the position of PSF in all three dimensions by looking up the z-ellipticity calibration curve (Fig. 1b). Through numerical simulation and experiments with fluorescent nanospheres and mammalian cells, we demonstrate that the proposed single-iteration algorithm can achieve localization precision close to multiple iterative GF based algorithm in all three dimensions, while yielding over 100 times faster computation speed.

## Results

The localization precision and execution speed of our gradient fitting based algorithm were compared with four commonly used localization methods, including QuickPALM^15^, nonlinear least squares Gaussian function fitting using width-difference calibration (NLLS-WD)^10^, nonlinear least squares Gaussian function fitting using width-approximation calibration (NLLS-WA)^5^, and maximum likelihood Gaussian function fitting using width-approximation calibration (MLE-WA)^19^,^20^. QuickPALM is a widely used single-iterative 3D localization algorithm for online localization imaging when computational simplicity is crucial. NLLS-WD and NLLS-WA are the most commonly used localization methods because of their high precision, robustness and relatively faster speed compared to MLE. MLE-WA is considered as the most precise method that achieves theoretically minimum uncertainty (Crámer-Rao lower bound, CRLB) to date, when the PSF model and noise model are correctly chosen.

### Localization precision and speed *via* numerical simulation

**Figure 2:**
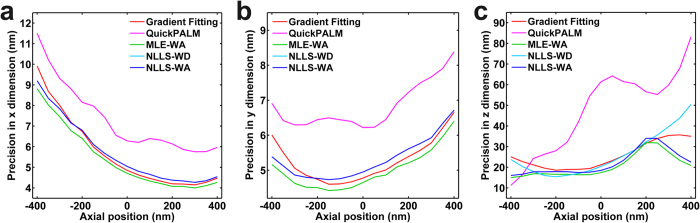
Comparison of localization precision using simulation. Localization precision in x dimension (**a**), y dimension (**b**), and z dimension (**c**) at different imaging depths. Note that the localization precision is quantified as the standard deviation of the estimated positions. Given the known position of the simulated image, we also compared the localization accuracy, or the root mean square error between the actual position and the estimated position using these five methods, which is shown in Supplementary Figure S1.

We first compared the localization precision of all five algorithms with numerically simulated images, as shown in Fig. 2. The simulated images were generated using the fluorescent properties of the commonly used fluorophore Alexa-647 (see “Methods” for details). We found that our gradient fitting based algorithm achieves a localization precision similar to the GF based algorithm (NLLS-WD, NLLS-WA, and MLE-WA) in both lateral (Fig. 2a–b) and axial dimensions (Fig. 2c), at different axial positions. In particular, the precision of our algorithm in x–y dimensions is superior to that of NLLS-WA and NLLS-WD and second only to the most precise MLE-WA for a long range of axial positions from −200 nm to 400 nm. On the other hand, our algorithm maintains the fast speed as the single-iterative algorithm such as QuickPALM, but with a much better precision, especially in the axial dimension. Overall, our gradient fitting algorithm achieves a lateral precision of less than 10 nm and an axial precision of less than 40 nm for super-resolution localization imaging at the presented axial depth range and signal level.

Next, we evaluated the localization speed of these algorithms by counting the average time consumed for one localization, as shown in Table 1. Not surprisingly, we found that the two single-iteration algorithms (gradient fitting and QuickPALM) run much faster than the three multiple-iteration algorithms (NLLS-WD, NLLS-WA, and MLE-WA). More specifically, our gradient fitting based algorithm runs more than 100 times faster than GF based methods (NLLS-WD, NLLS-WA, and MLE-WA). Note that, a GPU or multicores CPU were previously used for GF based algorithm to accelerate the execution speed by 10 ~ 100 times^19^,^20^,^21^, but our algorithm shows a superior localization speed even without using any high-performance computing hardware.

### Localization performance *via* experiments with fluorescent nanospheres

We evaluated the performance of our gradient fitting based algorithm in an experiment using fluorescent nanospheres. Using a commercial super-resolution localization microscopy system (N-STORM, Nikon Inc.), we captured three image stacks of single fluorescent nanospheres (100 nm diameter, TetraSpeck, Life Technologies) at the axial position of 160 nm, 0 nm and −160 nm, respectively. At each position, we captured 1000 images with a frame rate of 100 fps, and an average photon number of ~10,000 per localization. The localized positions of the fluorescent nanospheres were projected onto the X, Y and Z dimensions, and the Gaussian function was used to fit the localization profile in the three dimensions.

**Figure 3:**
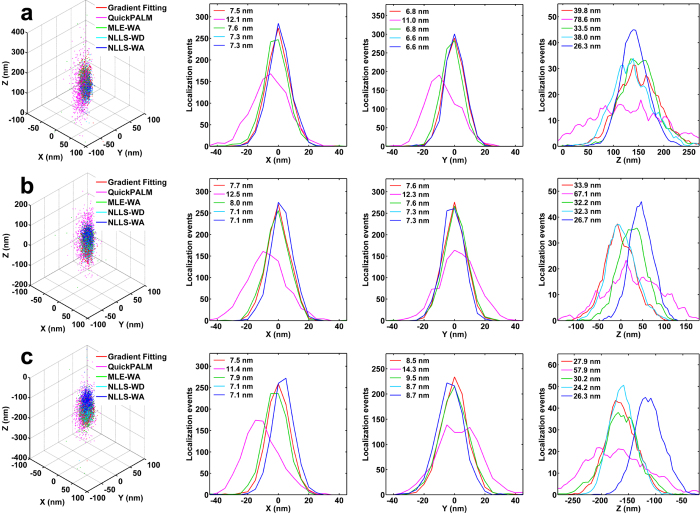
Localization performance of our gradient fitting based algorithm, QuickPALM, MLE-WA, NLLS-WA and NLLS-WD for experiments with fluorescent nanospheres. Localization precision is compared in X, Y and Z dimensions at the depth of 160 nm (**a**), 0 nm (**b**) and −160 nm (**c**). The localization precision of different algorithms is presented in the figure legend.

At three depths (160, 0–160 nm), our algorithm shows a precision of less than 11 nm in X and Y dimensions and less than 40 nm in Z dimension, close to that of the GF based algorithm (NLLS-WD, NLLS-WA, and MLE-WA), superior to the performance of QuickPALM, as shown in Fig. 3. This result is in good agreement with that of numerical simulation, although the precision of the experimental results of all five algorithms was a little worse, as system drift and unavoidable imaging aberration result in a non-perfect elliptical Gaussian function of the real PSF in the experiment. Our gradient fitting based algorithm, NLLS-WD and NLLS-WA provide the best performance in X and Y dimensions. However, the precision of the theoretically best MLE-WA is compromised due to the non-perfect Gaussian shape of the PSF. Note that, the deviation of the central z position between different algorithms comes from the different bias between the true position and the position derived from the corresponding calibration curve.

### Localization performance for 3D single-particle tracking

**Figure 4:**
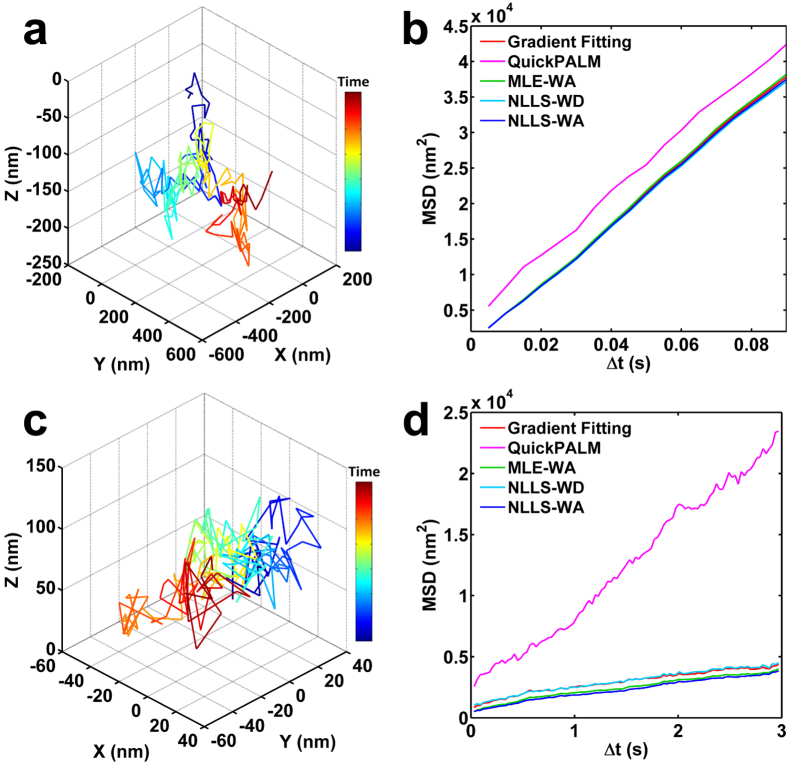
Localization performance in single-particle tracking experiments. The tracking trajectory of (**a**) a single fluorescent nanosphere and (**c**) a single telomere tracked by our gradient fitting based algorithm. Comparison of mean square distance (MSD) of (**b**) the nanosphere and (**d**) telomere movement for different localization algorithms.

We then evaluated the performance of our gradient fitting based localization algorithm in single-particle tracking for nanospheres and telomeres. First, we tracked a single fluorescent nanosphere (100 nm diameter, TetraSpeck, Life Technologies) in glycerol at a frame rate of 200 fps for one second and a photon number of ~10000 per localization. We compared the mean square distance (MSD) of the fluorescent nanosphere for different localization algorithms, and found that our gradient fitting based methods exhibit similar results with the GF based method (NLLS-WD, NLLS-WA, and MLE-WA), as shown in Fig. 4b. Second, we evaluated their performance in biologically significant telomere tracking^22^. Telomeres labeled with RFP in live U2OS cells were tracked for 6 seconds at a frame rate of 33 fps and a photon number of ~5000 per localization. Our algorithm also showed a similar MSD value to those from GF based methods (NLLS-WD, NLLS-WA and MLE-WA), as shown in Fig. 4c. As the exact position of the telomere is unknown, we cannot determine which algorithm provides the most accurate result. Nevertheless, the single-particle tracking experiment demonstrate that our gradient fitting based algorithm gives similar particle-tracking results (MSD) with the GF based method (NLLS-WD, NLLS-WA, and MLE-WA) in both ideal physical sample and live mammalian cells.

### Localization performance for 3D STORM imaging

**Figure 5:**
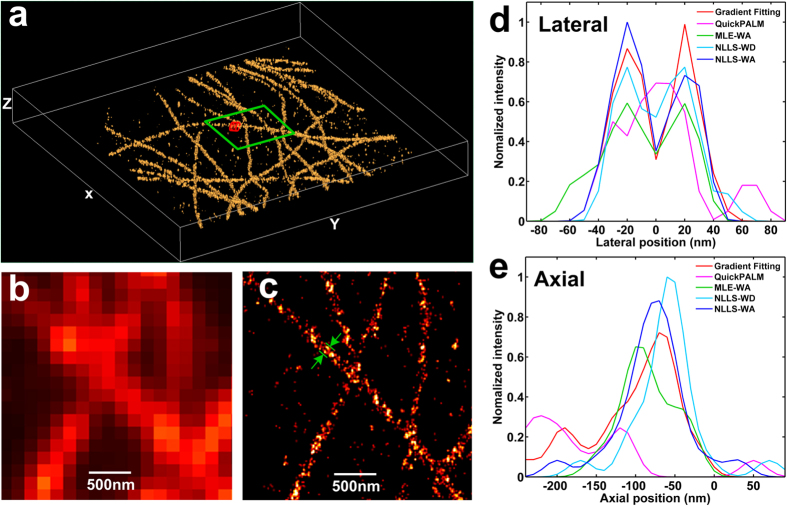
3D STORM imaging of microtubules in MEF cells. (**a**) The 3D STORM image reconstructed by our gradient fitting based algorithm. (**b,c**) The higher zoom of (**b**) the conventional wide-field image and (**c**) the lateral plane projection of STORM image for the area shown in the green box of (**a**). Localization performance of different algorithms are compared in lateral dimension (**d**) and axial dimension (**e**). Scale bar: 500 nm.

We also evaluated the performance of our gradient fitting based algorithm in 3D super-resolution localization imaging of microtubules. The microtubules labeled with Alexa-647 in fixed mouse embryo fibroblast (MEF) cells were imaged with N-STORM (Nikon) and reconstructed using our gradient fitting algorithm, as shown in Fig. 5a,c. Figure 5d,e show the molecule counting distribution profile in lateral and axial dimensions, to characterize the localization performance from five different localization algorithms. We found that our algorithm yields a lateral full width at half maximum (FWHM) of ~35 nm (Fig. 5d) in imaging microtubules of MEF cells, which can clearly resolve the hollow structure (~40 nm between the two peaks); and an axial FWHM of ~90 nm (Fig. 5e), close to that from GF based algorithm (NLLS-WD, NLLS-WA, and MLE-WA), and much better than that from QuickPALM, consistent with our simulation study. Note that, the average photon number per localization events is ~1500 in this experiment, which represents non-ideal scenario without a full optimization of photon efficiency. To localize all the molecules (~30,000) of this experiment (Fig. 5a), our algorithm consumed ~2.7 seconds. Compared to over 6.5 minutes using GF based algorithms, it represents more than 100 times improvement, consistent with our previous simulation results.

## Discussion

We present an efficient 3D localization algorithm based on gradient fitting for single-particle tracking or super-resolution localization microscopy with both superior localization precision and execution speed. Compared to traditional GF based algorithm, this single-iteration algorithm is based on simple algebraic operation without significant computational complexity, and thus can be implemented with a high computational speed. Unlike the widely used single-iteration localization algorithm—QuickPALM that compromises precision for high speed, our gradient fitting based algorithm archives a similar precision to those multiple iteration GF localization algorithms, but is still competitive in localization speed. Therefore, our gradient fitting based algorithm represents a promising approach for high-speed implementation of 3D super-resolution image reconstruction and single-particle tracking in embedded devices^23^ and low-cost, portable devices such as smart phones^24^,^25^. Moreover, considering that the high-speed imaging cameras, such as scientific Complementary Metal Oxide Semiconductors (sCMOS), has been introduced to super-resolution localization microscopy with a large field of view^26^,^27^, the speed advantage of our algorithm will also be more attractive for high-throughput 3D super-resolution imaging and single-particle tracking. Alternatively, in the case where multiple iteration GF localization algorithm is still required, our algorithm can be used for the first iteration to get a more accurate estimation of the starting point and significantly reduce the iteration times. If a more appropriate gradient operator, weighting parameter and initial values can be identified, the performance of this method will be further improved. Further, given that no symmetric shape of the PSF is assumed, this method, in principle, is capable of retrieving the position for any kind of PSF. Although we only demonstrated the 3D position retrieval capability of gradient fitting based method, we believe gradient fitting can also be used in other applications, *e.g*. molecule dipole orientation detection, pattern matching *etc*. by combining *a priori* information of PSF.

## Conclusion

In conclusion, we describe a single-iterative localization algorithm for 3D single particle tracking and super-resolution localization microscopy using astigmatism imaging. Our algorithm employs gradient distribution fitting to determine the precise 3D position of the fluorescent emitter. We demonstrate that our gradient fitting based algorithm is capable of reconstructing 3D super-resolution images with a precision similar to the standard interactive Gaussian function fitting algorithm across a wide range of depth, while executing at more than 100 times faster. We believe that this algebraic algorithm has a great potential for high-speed online data analysis of 3D super-resolution localization microscopic images in embedded devices and low-cost and portable microscopy using smart phones.

## Methods

### Gradient fitting based algorithm

Generally, the emitter’s PSF profile (*I*) in the astigmatism-based microscopy can be approximated by a 2D elliptical Gaussian function, which can be expressed by the following equation:^5^
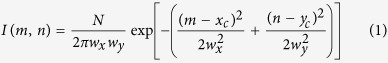
where (*x*_c_, *y*_c_) is the lateral center position of the emitter, (*w*_x_, *w*_y_) is the width of the emitter’s PSF in *x* and *y* dimensions, N is the emitter’s total photon numbers and (*m*, *n*) is the coordinate on the lateral plane.

By calculating the partial derivatives of the above elliptical PSF, the emitter’s exact gradient distribution (G) can be easily derived as follows:
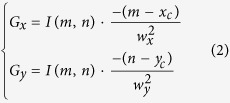
For a noise-free, non-pixelated image, the measured gradient distribution of the PSF should be equal to its exact value G. But, given the unavoidable shot noise and finite pixel size, the measured image is a noisy and pixelated image, and the exact G cannot be directly obtained. In this case, we first use two optimized gradient operators to convolve with the raw image (*A*) to get the measured gradient distribution (*g*), which gives a good approximation of the exact value *G*:
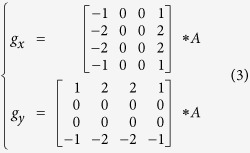


However, the measured gradient distribution *g* may still deviate from its exact value *G*. Hence, we utilize the nonlinear least squares method to find the best-fit *G* with the minimal total deviation (*D*) to the measured *g*. The deviation is defined as the angle (*θ*) between *G* and *g*, which can be approximated by the following equation:
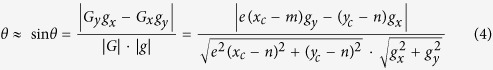


where *e* is defined as the ellipticity (*w*_*y*_/*w*_*x*_)^2^. Note that, *θ* can be approximated by sin *θ*, given small *θ*.

Then, the total deviation (*D*) can be determined as the sum of the *θ*^2^:
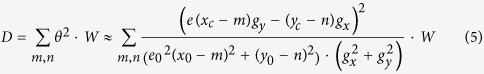


where *e*_0_ and (*x*_0_, *y*_0_) are the initial value of *e* and (*x*_*c*_, *y*_*c*_), which are pre-estimated by the centroid method^15^. *W* is the weighting parameter^18^, considering that the gradient direction is more accurately determined (i.e., larger *W*) at positions with higher intensity-gradient or lower intensity variation. In other words, at the positions near the center of emitter, a higher intensity variation is often seen (due to the higher intensity level of the emitter), which results in a smaller weighting parameter. This weighting parameter *W* can be simply defined by the following equation:
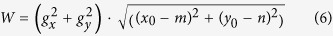


Note that, employing only the gradients in the central area for analysis improves the performance of this algorithm. Because the signal of the outer pixels are relative low, they are more easily affected by the background and the signals from the neighboring emitter, which leads to obvious localization errors.

Mathematically, *D* achieves the minimal value at the position where the partial derivative is equal to zero, and the estimated lateral center position and ellipticity *e* could be obtained by a closed-form solution of the following equation:
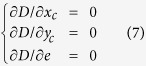


Then, we need to decode the axial position. Two calibration methods are generally used: width-difference calibration for smaller bias and width-approximation calibration for better precision^28^. Width-difference calibration determines the unknown axial position by looking up the width-difference (*w*_*y*_–*w*_*x*_) calibration curve^15^, while width-approximation method estimates the unknown axial position by comparing (*w*_*x*_, *w*_*y*_) with two width-calibration curves and finding the best-fit value^5^. Here, because our algorithm directly calculates the ellipticity *e*, so we use *z*–*e* calibration curve for our algorithm to retrieve the unknown z position.

Finally, starting from the raw images, the complete procedures of our gradient fitting based algorithm for 3D localization of single fluorophore are summarized as follows.

Step 1: Denoising and fluorophore extraction as described previously^15^,^20^.

Step 2: Determine the *x*–*y* position and ellipticity *e* of the emitter’s PSF using eq. (3, 4, 5, 6, 7).

Step 3: Estimate the *z* position according to the *z*–*e* calibration curve.

The source code implemented in Matlab can be found on our website: http://www.pitt.edu/~liuy.

### Axial position calibration

We acquired z-stack images of a single fluorescent nanosphere (100nm diameter, TetraSpeck, Life Technologies) at a series of different axial positions controlled by a nano-positioning stage and MLE-WA algorithm was used to retrieve the Gaussian kernel (*w*_*x*_, *w*_*y*_) for each z position, to build the calibration curve (as shown in Supplementary Figure S2). The position with the minimal width difference (|*w*_*x*_–*w*_*y*_|) is defined as the zero position. Please note that four images were acquired at each axial position and the average value of *w*_*x*_, and *w*_*y*_ were used at each position to reduce the deviation. A 4^th^-order polynomial is used to fit each calibration curve.

### Numerical simulation

To evaluate the performance of our gradient fitting based method, a series of image sets with a single molecule were numerically generated. The molecule was randomly distributed in the central pixel of a 33 × 33 pixels image. The PSF was modeled with integrated elliptical Gaussian function, and the width of Gaussian kernel (*w*_*x*_, *w*_*y*_) was set according to the value retrieved from the calibration curve using fluorescent nanospheres (Supplementary Figure S2), with a defocus depth from −0.4 μm to 0.4 μm. The pixel size was set to be 160 nm to match the experimental setup. The total photon number of the molecules were kept to be 5000 photons to mimic the photon number of a commonly used fluorophore (Alexa 647) in the experiment^29^. The background was set to be 100 photons per pixel to be consistent with the experimental dataset. The noise was modeled with a Poisson model, considering a low-light detector with negligible camera noise typically used to capture the image. For each axial position, 1000 images were generated and analyzed. Subregion of 15 × 15 pixels were extracted for all algorithms.

### Optical system

Commercial microscopy from Nikon Instruments (N-STORM) was employed for all imaging and tracking experiments in this paper. Under the oblique angle illumination, the fluorescent emission was collected by the objective (100×, NA 1.49, oil immersion, Nikon), together with the EMCCD camera (iXon 897, Andor), and a cylindrical lens is inserted in the optical path for 3D astigmatism imaging. A Perfect Focus System (PFS) was used in all the experiment for dynamic drift correction. All analysis were performed using MATLAB R2014a (MathWorks) on the same desktop computer (Intel Core i7-4790, 3.60 GHz). The 3D image in Fig. 5 is reconstructed using Volview 3.4.

### 3D telomere tracking

U2OS cells were cultured in Dulbecco’s Modified Eagle’s Medium (DMEM, Lonza) with 10% fetal bovine serum (Atlanta Biologicals) at 37 °C and 5% CO_2_. Before imaging, cells were transfected with RFP-TRF1 for 24 hours. RFP-TRF1 was bound to telomeric DNA, serving as a surrogate marker for telomeres. A 561 nm excitation laser was used and the telomeres were tracked for 6 seconds with an exposure time of 30 ms and EM gain of 10.

### 3D Super resolution localization imaging

MEF cells were used for 3D super resolution localization imaging. MEF cells were planted on a glass bottomed petri dish and cultured in Dulbecco’s Modified Eagle’s Medium (DMEM, Lonza) with 10% fetal bovine serum (Atlanta Biologicals) at 37 °C and 5% CO2 for 24 hours before immunostaining. Then, the cells were fixed with 1:1 acetone/methanol solution for 10 minutes at room temperature, followed by standard immunofluorescence staining procedure. In brief, the cells were incubated with Anti-alpha tubulin rabbit primary antibody (abcam) overnight at 4 °C and 2 h with Alexa 647 F(ab′)2-goat anti-mouse/rabbit IgG (H + L) secondary antibody (Invitrogen). Immediately before imaging, the buffer was switched to the STORM imaging buffer according to Nikon N-STORM protocol (50 mM Tris-HCl pH 8.0, 10 mM NaCl, 0.1 M cysteamine (MEA), 10% w/v glucose, 0.56 mg/mL glucose oxidase, 0.17 mg/mL catalase). In this experiment, 647 nm laser was used for excitation, and we acquired 20,000 frames with exposure time of 60 ms and EM gain of 100. Note that, additional drift correction algorithm based on cross-correlation was used for accurate system drift correction.

## Additional Information

**How to cite this article**: Ma, H. *et al*. Fast and Precise 3D Fluorophore Localization based on Gradient Fitting. *Sci. Rep*. **5**, 14335; doi: 10.1038/srep14335 (2015).

## Supplementary Material

Supplementary Information

## Figures and Tables

**Figure 1:**
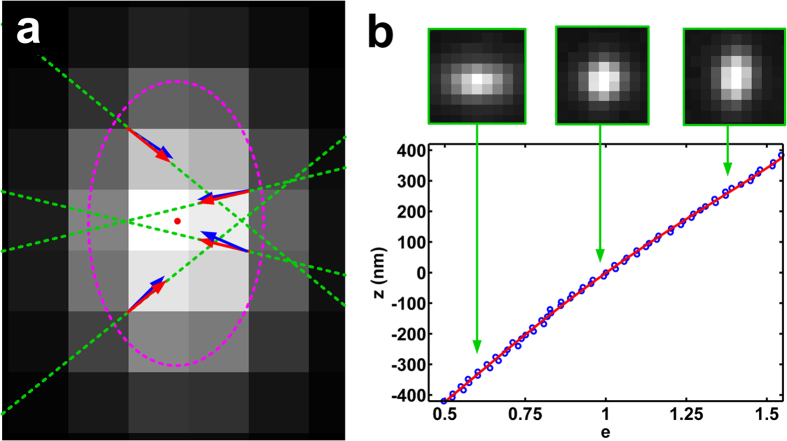
The principle of the gradient fitting based algorithm. (**a**) The image of a single fluorescent emitter, where the red dot indicates the exact x–y position of the molecule, the red and blue arrows show the exact gradient directions and the calculated gradient directions of that position, respectively; the green dashed lines indicate the corresponding exact gradient lines, and the magenta dashed ellipse indicates the shape of the PSF. (**b**) The z–e (ellipticity) calibration curve used to look up the axial position according to the calculated ellipticity. Three representative patterns of a single emitter are shown to indicate the PSFs at the corresponding axial positions. Note that a 4th-order polynomial function is used to fit the z–e calibration curve.

**Table 1:** Average time consumption per localization for the simulated images.

Method	Simulation	
	Time (ms)^a^	Gain^b^
Gradient fitting	0.089	1
QuickPALM	0.025	0.28
MLE-WA	18.3	206
NLLS-WD	12.4	139
NLLS-WA	12.9	145

^a^Averaged from 10,000 simulated molecules with a sub-region box of 15 × 15 pixels for all the five algorithms.

^b^The speed gain of our gradient fitting based algorithm over other algorithms.
