# Supplementary material for: Fast and Precise 3D Fluorophore Localization based on Gradient Fitting
Source: Sci Rep. 2015 Sep 22;5:14335. doi: 10.1038/srep14335 (PMC4585720; doi:10.1038/srep14335)
Supplement: Supplementary Information [file srep14335-s1.pdf]

Supplementary Information

Fast and Precise 3D Fluorophore Localization based on Gradient Fitting

Hongqiang Ma[1], *Jianquan Xu*[1], *Jingyi Jin*[1,2], *Ying Gao*[2,3], *Li Lan*[3] *and Yang Liu*[1]*

[1]Biomedical and Optical Imaging Laboratory, Departments of Medicine and Bioengineering, University of Pittsburgh, Pittsburgh PA 15213, USA
[2]School of Medicine, Tsinghua University, China
[3]Department of Microbiology and Molecular Genetics, University of Pittsburgh, Pittsburgh PA 15213, USA
*liuy@pitt.edu

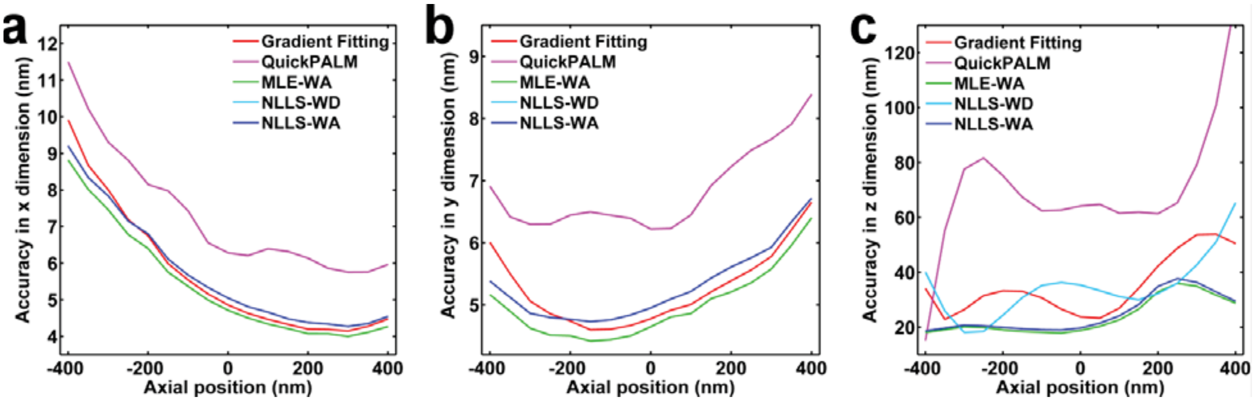

Figure S1. Comparison of localization accuracy using simulation. Localization accuracy in x dimension (a), y dimension (b), and z dimension (c) at different imaging depths. Note that the localization accuracy in simulation is quantified as the root mean square error between the estimated position and the corresponding true position.

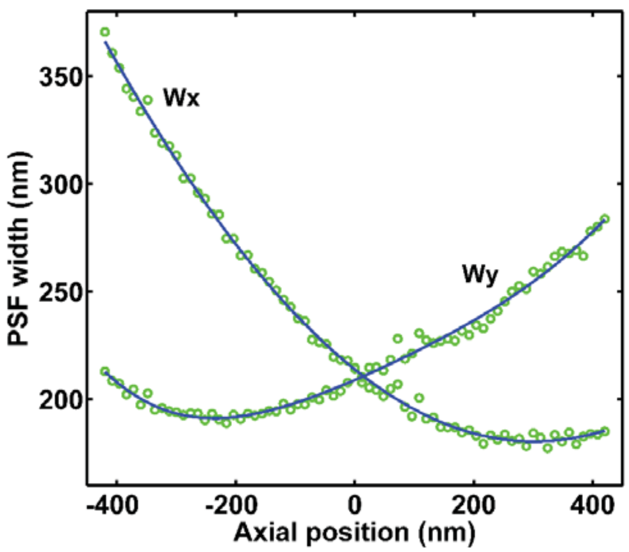

Figure S2. Calibration curve of point spread function (PSF) widths w_x and w_y as a function of axial position.
